# Combining Forces - The Use of Landsat TM Satellite Imagery, Soil Parameter Information, and Multiplex PCR to Detect *Coccidioides immitis* Growth Sites in Kern County, California

**DOI:** 10.1371/journal.pone.0111921

**Published:** 2014-11-07

**Authors:** Antje Lauer, Jorge Talamantes, Laura Rosío Castañón Olivares, Luis Jaime Medina, Joe Daryl Hugo Baal, Kayla Casimiro, Natasha Shroff, Kirt W. Emery

**Affiliations:** 1 Department of Biology, California State University, Bakersfield, California, United States of America; 2 Department of Physics & Engineering, California State University, Bakersfield, California, United States of America; 3 Laboratorio de Micología Médica, Facultad de Medicina, Universidad Nacional Autónoma de México, Mexico City, Mexico; 4 County of Kern Public Health Services Department, Bakersfield, California, United States of America; California Department of Public Health, United States of America

## Abstract

Coccidioidomycosis is a fungal disease acquired through the inhalation of spores of *Coccidioides* spp., which afflicts primarily humans and other mammals. It is endemic to areas in the southwestern United States, including the San Joaquin Valley portion of Kern County, California, our region of interest (ROI). Recently, incidence of coccidioidomycosis, also known as valley fever, has increased significantly, and several factors including climate change have been suggested as possible drivers for this observation. Up to date details about the ecological niche of *C. immitis* have escaped full characterization. In our project, we chose a three-step approach to investigate this niche: 1) We examined Landsat-5-Thematic-Mapper multispectral images of our ROI by using training pixels at a 750 m×750 m section of Sharktooth Hill, a site confirmed to be a *C. immitis* growth site, to implement a Maximum Likelihood Classification scheme to map out the locations that could be suitable to support the growth of the pathogen; 2) We used the websoilsurvey database of the US Department of Agriculture to obtain soil parameter data; and 3) We investigated soil samples from 23 sites around Bakersfield, California using a multiplex Polymerase Chain Reaction (PCR) based method to detect the pathogen. Our results indicated that a combination of satellite imagery, soil type information, and multiplex PCR are powerful tools to predict and identify growth sites of *C. immitis*. This approach can be used as a basis for systematic sampling and investigation of soils to detect *Coccidioides* spp.

## Introduction

Valley fever research has predominantly focused on the medical and epidemiological aspects of *Coccidioides immitis* and *Coccidioides posadasii*, the fungi that cause coccidioidomycosis ([1], [2], and references therein). *Coccidioides* spp. can have a complete life cycle as soil dwelling organisms but if the soil is disturbed, their arthroconidia can become air-borne and are able to infect a host via the respiratory tract. About 60% of infected patients report no symptoms [3]; about 25% exhibit severe flu-like symptoms, such as cough, sputum, fever, and muscle aches; the remaining 15% become very ill with pneumonia-like symptoms (e.g. pleurisy and heavier sputum) requiring medication and bed rest. In a small number of cases (about 0.5–1%), the disease disseminates beyond the lungs to e.g. the skin, bones, and/or meninges of the brain, and the disease can be fatal. Certain sectors of the population seem to be more susceptible to infection, such as the very young, persons newly arrived to the endemic areas (since immunity develops with infection), field-, and construction workers, and those with impaired immune systems [4].

*Coccidioides* spp. are endemic in the southern part of the San Joaquin Valley in California, southern California, the southern part of Arizona, New Mexico and Texas, most of northern Mexico, and some areas in Guatemala, Honduras, Venezuela, northeastern Brazil, Argentina, and Paraguay [5], [6]. Given its geographic distribution, it is evident that *C. posadasii* is able to flourish in desert regions of the Americas (besides California), in contrast to its close relative *C. immitis* which seems to be restricted to areas in California. Of the two fungal species, it is *C. immitis* which afflicts the San Joaquin Valley portion of Kern County, California [7], [8] which is the Region of Interest (ROI) of this study. However, population genomic sequencing of *Coccidioides* spp. revealed recent hybridization between both species [9], and nothing is known about the distribution and ecology of these hybrids.

It is reasonable to expect that climatic fluctuations might affect the rate at which humans become infected [4]. For example, an extended drought might decimate less heat tolerant, non-sporeforming soil microorganisms that had acted as natural antagonists to the pathogen in its natural environment. A wetter than-normal rainy season could help *Coccidioides* spp. bloom, and windy spells might facilitate the dispersal of its arthroconidia. The “grow and blow” hypothesis has first been introduced by Comrie and Glueck [10]. It has long been surmised that *Coccidioides* spp. are generally poor competitors [11], but that they are more heat-resistant than competing microorganisms – thus, it can be expected that hot summers might favor its presence or dominance. Indeed, anecdotal evidence to these effects is well documented in the literature [12]–[20]. There have been a number of attempts at demonstrating this connection quantitatively with various degrees of success [10], [11], [21]–[25]. Yet, despite extensive study, there is currently no ecologically consistent link identified between the environment and coccidioidomycosis rates [26]. The predicted warming of the climate in California will add another piece of the puzzle in the already complicated interrelationships of environmental factors that might support or suppress the growth of pathogens with environmental reservoirs [27], [28]. However, occasionally, the pathogen was detected in other regions, such as the recent detection of *Coccidioides immitis* in soils of Eastern WA [29].

There also have been several attempts to characterize the ecological niche of *Coccidioides* spp. in more detail [4], [31]–[33], but we still do not have a complete description of this niche. To date, Fisher et al. [31] present the most comprehensive review of this subject. We need to direct attention to a few fundamental points about what is known in regards to this niche. First, it is important to realize that *C. immitis* and *C. posadasii* do not grow in disturbed soils [4], [30], [31] such as cultivated fields, gardens, etc. Second, whereas it was initially thought that *Coccidioides* spp. ecological niche corresponds to the Lower Sonoran Life Zone (as defined and described by Merriam [34]), or similar environments [14], [35], [36]. Later research [30] showed that this is not quite correct, and indeed more recent works [31], [32], [37], [38] suggested that the fungus's niche corresponds more closely with thermic and hyperthermic soils in which temperatures can reach or exceed 22°C in 50 cm depth. Fisher et al. [31] described sites where *Coccidioides* spp. were suspected to have been present because humans or animals were reported to have been infected at these sites. Fisher et al. [31] also made the general observations that the vegetation at those sites ranged from sparse to relatively thick cover in lower Sonoran Deserts, Chaparral-upper Sonoran brush and grasslands, as well as Mediterranean savannas and forested foothills. Furthermore, they stated that the temperature regimes, climate conditions in general, and soil textures are the only indicative variables of the presence of *Coccidioides* spp. Microbial diversity in soils is highly influenced by the habitat's chemical and physical parameters. But biotic soil factors such as plant and microeukaryote diversity influence fungal and bacterial soil communities as well through root exudation (additional available nutrients), microbial antagonism (antibiotic production) and synergism, as well as through selective grazing by microeukaryotes [32], [39]–[40]. It is currently being discussed that the pathogen is in fact not very competitive as a soil saprophyte because it has lost the ability to produce a variety of enzymes that are involved in important biodegradation processes of soil organic matter, which might explain the difficulty to detect it in bulk soil [41].

There are few published data available about the distribution of *C. immitis* growth sites in Kern County, California [42] most probably because it has been very difficult in the past to isolate and identify *Coccidioides* spp. from soil and dust samples [4], [14], [30]. Recently, first attempts using molecular biological techniques to identify *C. immitis* in bulk soil samples from Kern County, predominantly around Bakersfield, have been performed [32]. Based on that study, it appeared that *C. immitis* is likely to be found in the Bakersfield area at locations that are non-agricultural and have about equal parts of sand, clay, and silt (clay loam), a pH between 7.8 and 8.5, an available water capacity of about 0.15–0.2 cm/cm, a water content of about 30% (1/3 bar), an available water supply (0–25 cm) of 4–5 cm, and a Cation Exchange Capacity (CEC7) of over 20 milliequivalents per 100 grams.

The idea of using remote sensing (RS) techniques to piece together environmental characteristics, environmental change, and their relationship to disease transmission has been used extensively in connection with other diseases such as malaria [43], cholera [44], and African trypanosomiases [45]. Even though the ecological niche of *C. immitis* is not well characterized, we present here a RS technique that allows the mapping of sites around Bakersfield, California, where the pathogen is suspected to grow based on data obtained in a previous study by Lauer et al. [32]. Our method utilized a location well-known for being a *C. immitis* growth site (Sharktooth hill [STH], Bakersfield, California) as a basis, and then examined satellite images of the ROI to find all locations with similar spectral signatures. This is similar to characterizing the growth sites by the vegetation that tends to grow in the same environment as *C. immitis*, using the vegetation type as a marker. This is reasonable because the vegetation type closely reflects the co-variation of the relevant physical and chemical parameters such as clay and sand content, temperature, pH, nutrients, water content, etc., and also affects the development of the microbial diversity in the soils [46].

To validate our approach, we investigated if a combination of remote sensing and soil parameter information can predict locations which might be suitable to support the growth of *C. immitis*, followed by a molecular biological approach to detect the fungus in these soils with a culture independent polymerase chain reaction (PCR) based method [32], [47].

## Material and Methods

No specific permissions were required for the soil sampling. Our field study did also not involve endangered or protected species.

### Multispectral image analysis

Landsat-5 Thematic Mapper (TM) L1G corrected multispectral images were downloaded from the United States Geological Survey archive (http://EarthExplorer.usgs.gov). The satellite relayed a continuous data stream which was then framed into individual scenes each 23.92 sec (see, e.g., http://landsat.gsfc.nasa.gov/about/wrs.html). The images for path 42, rows 35 and 36: Worldwide Reference System to cover our ROI were downloaded, and then the two images were mosaicked. Most of the analysis that is presented here was performed on a spatial subset of this mosaicked image. This subset corresponds to an area approximately one million hectares that covers the San Joaquin Valley portion of Kern County. Our work mainly focused on a multispectral image taken on April 20, 2008 at 10:23 PM local time. This image was chosen because it was obtained at a date (during spring) were microbial activity and biomass in the soil is generally considered high, because of supportive environmental parameters, such as moderate temperatures and increased water content. Furthermore, this image had 0% cloud cover. Our analysis started by defining a 25 pixel ×25 pixel area centered at latitude 35° 28′ 20.29′′ N, and longitude 118° 54′ 37.04′′ W. This location is at STH, an area where *C. immitis* has been repeatedly detected ([30], [31], [48], this study). These 625 pixels were used to train the algorithm, and thus, they define a spectral class which is referred to in what follows as the “STH-vegetation class”. To implement the Maximum Likelihood Classification (MLC) method distributed (Richards & Jia, 2006), TM bands 1 (0.45–0.52 µm, blue-green), 2 (0.52–0.60 µm, green), 3 (0.63–0.69 µm, red), 4 (0.76–0.90 µm, near infrared), 5 (1.55–1.75 µm, mid infrared), and 7 (2.08–2.35 µm, mid infrared) were used. Band 6 (10.40–12.50 µm, thermal infrared) was not used in our MLC scheme because the resolution was 60 m instead of the 30 m (as it is for the other bands). However, this band was used to compute surface temperatures as described in more detail below. Our MLC scheme then entailed computing, for each of the pixels in the ROI, the probability that it belonged to the STH-vegetation class. This probability was assumed to be normally distributed [49], and thus is given by
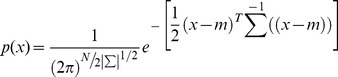



where ***x*** is a vector location in pixel space, *N = 6* is the dimensionality of pixel space, **Σ** the covariance matrix of the distribution, and ***m*** is the mean position of the spectral class. (***m*** and Σ are computed from the training pixels). A threshold value was set at *p_0_*, meaning that if a pixel had a probability 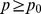 of being in the STH-vegetation class, then the pixel was put into this spectral class. Otherwise 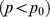 the pixel was simply left unclassified. Clearly, as the parameter *p_0_* decreased, the fraction *f* of pixels in the ROI which belong to the STH-vegetation class increased. This is because pixels which are less and less like the training pixels get included into this class.

It was also investigated how much *p_0_* needed to be reduced from 1 until the sites which tested positive for *C. immitis* came into the STH-vegetation class. This served to calibrate the method and as a validation step. Clearly, if the *C. immitis*-positive sites get included in the STH-vegetation class for 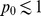, then our method is robust. However, if our *C. immitis*-positive sites remain unclassified until 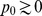, then our spectral class is poorly defined.

Lastly, we determined the area (km^2^) that was characterized by vegetation that belonged into the STH-vegetation class over the sampling period and until early 2014 using landsat images and the software ENVI 5.1+IDL 8.3.

### Surface temperatures

Surface temperature variations across the ROI were of interest as well. In addition to utilizing a vegetation class to assess potential sites for *C. immitis* growth, surface temperature variations across the ROI may also help to characterize the niche of this fungus. Landsat-5 TM-6 is an infrared band. This band (from the same April 20, 2008 image) was used as follows to examine the thermal landscape of our ROI. The same area in STH was taken as training pixels, and their average 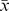 and standard deviation 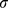 were computed. Then, a simple parallelepiped method [49] was used to find other locations in the ROI with similar values. Thus, all pixels whose value was in the ranges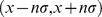, *n = 1,2,3* were put into this spectral class, and we referred to this as the “STH-thermal” class. All other pixels were left unclassified. The image's digital numbers were converted to temperatures by applying the procedure described in the National Aeronautics and Space Administration's Landsat 7 Science Data Users Handbook (http://landsathandbook.gsfc.nasa.gov/). See also Chander & Markham [50] for details. As a result, a map was obtained where the surface temperature was close to STH at the time the image was taken.

### Weather data

Precipitation data for the Southern San Joaquin Valley was obtained from the California Data Exchange Center (http://cdec.water.ca.gov/snow_rain.html). The cumulative monthly precipitation (inches) over time was assembled from 5 stations (Calaveras Big Trees [CVT], Hetch Hetchy [HTH], Yosemite HQ [YSV], North Fork RS (NFR), and Huntington Lake (HNT]). A more detailed analysis of the weather data for Bakersfield in particular was not the focus of this study.

### Physical and chemical soil parameters

To determine physical and chemical soil parameters of all soil samples, the websoilsurvey database of the United States Department of Agriculture (http://websoilsurvey.nrcs.usda.gov/) was used. Furthermore, all sampling sites were characterized by using the soil series extent mapping tool from the website of the Center of Environmental Informatics (CEI) (http://www.cei.psu.edu/cei_wp/). Thus, through agricultural and environmental support tools available from the USDA and CEI websites, our sampling sites were further characterized in regard to land use and vegetation. Additional geological information was obtained as well, such as the distribution of certain soil types and series in California. By using the soil series extent mapping tool, our soil samples were linked to known soil series and soil groups that are characteristic for the Southern San Joaquin Valley and beyond.

### Soil sampling sites

Soil physical and chemical parameters that could likely support the growth of the pathogen based on results of the study by Lauer et al. [32] were used to choose 13 new sites that were investigated in winter and spring 2011 (Jan–Apr). Additionally, two sites that were found to be strong growth sites of the pathogen in 2008/2009 were also investigated again in 2011. Six sites were the pathogen was not detected were included in this study as well. Sampling sites included in this study were all non-agricultural silt, clay or sandy loams that differed in regard to physical and chemical parameters. All sites were located within the Central Valley Portion of Kern County. Overall, 23 sites were investigated in this study by satellite imagery and multiplex PCR. Two additional sites from STH were investigated by satellite imagery only (reference sites). Based on information from the USDA websoilsurvey database, the soils belonged to 13 different soil map units. Samples were taken each month in 2008, 2009 and 2011 (some sites were not sampled in 2011) from three different depths (0–2 cm, 5–7 cm, and 18–20 cm), placed on ice during transport to the lab, and frozen at −80°C when not processed immediately. See table 1 for detailed information about all sites, including exact location, soil type, observed rodent activity and indication of the presence or absence of *C. immitis*. Also see the first column of table two for the year they were investigated. Our sampling sites were not chosen based on Landsat imagery. They were chosen mainly based on the percentage of clay in the soil as indicated by the USDA websoilsurvey database. About 30% of clay had been indicative of a potential *C. immitis* positive site based on previous research [32]. After results from the multiplex PCR approach became available, we evaluated if sites where *C. immitis* was detected correlate with sites indicated by Landsat imagery to fall into STH vegetation sites.

**Table 1:** Location and description of sampling sites used as test data for the remote sensing approach.

sampling sites and year sampled	coordinates	soil type (map unit symbol)	GS or AS of the pathogen	rodent activity*
**Bakersfield city**				
1. CSUB Children Center (′08, ′09)	119° 06′ 29.0′′ W, 35° 20′ 57.0′′ N	Wasco sandy loam (243)	AS	yes
2. Belle Terrace/P Str. (‘11)	119° 00′ 37.2′′ W, 35° 20′ 49.8′′′ N	Kimberlina Urban land, Cajon-complex (180)	GS	no
3. Belle Terrace/Gay Str. (‘11)	118° 59′ 22.7′′ W, 35° 20′ 40.0′′′ N	Kimberlina Urban land, Cajon-complex (180)	GS	yes
4. Marella Way (‘11)	118° 63′ 15.0′′ W, 35° 21′ 40.0′′′ N	Kimberlina Urban land, Cajon-complex (180)	NS	no
5. Flood Plain CSUB (′08, ′09)	119° 06′ 05.0′′ W, 35° 21′ 16.0′′ N	River Wash (229)	AS	no
**SW Bakersfield**				
6. Bike Path West (′08, ′09)	119° 15′ 06.0′′ W, 35° 18′ 20.0′′ N	Cajon sandy loam (125)	NS	yes
7. Lake Webb (′08, ′09)	119° 16′ 27.0′′ W, 35° 13′ 53.0′′ N	Zalvidea sandy loam (240)	AS	no
8. Cole's Levee Rd. I (′08, ′09, ‘11)	119° 13′ 60.0′′ W, 35° 14′ 08.0′′ N	Garces loam (180)	GS	yes
9. Cole's Levee Rd. II (‘11)	119° 13′ 65.3′′ W, 35° 14′ 09.7′′ N	Garces loam (180)	GS	yes
10. Olen Avenue (‘11)	119° 14′ 50.0′′ W, 35° 14′ 72.0′′ N	Garces loam (180)	GS	yes
11. Valley Street Field (′08, ′09)	118° 52′ 18.0′′ W, 35° 24′ 29.0′′ N	Delano sandy loam (139)	AS	no
**NE Bakersfield**				
12. Across CALM (‘11)	118° 53′ 14.1′′ W, 35° 25′ 50.3′′ N	Chanac Clay Loam (130)	GS	yes
13. Ant Hill Oil Field (′08, ′09, ′11)	118° 51′ 25.0′′ W, 35° 23′ 50.0′′ N	Chanac Clay Loam (131)	GS	yes
14. Round Mt. Rd. I (′08, ′09)	118° 52′ 20.0′′ W, 35° 27′ 10.0′′ N	Xeric Torriorthents-Calcic Haploxerept association (174)	AS	yes
15. Round Mt. Rd. II (′08, ′09)	118° 53′ 30.0′′ W, 35° 28′ 42.0′′ N	Xeric Torriorthents-Calcic Haploxerept association (174)	NS	yes
16. Sharktooth hill I	118° 55′ 03.4′′ W, 35° 27′ 44.5′′ N	Chanac Pleito Premier Association (305)	nd	yes
17. Sharktooth hill 2	118° 54′ 37.0′′ W, 35° 28′ 20.0′′ N	Pleito Trigo Chanac Complex (205)	GS	yes
18. Sharktooth hill 3	118° 54′ 33.0′′ W, 35° 28′ 21.3′ N	Pleito Trigo Chanac Complex (205)	GS	yes
**NW Bakersfield**				
19. Acari Rd. (‘11)	119° 15′ 26.8′′ W, 35° 23′ 16.1′′ N	Garces silt loam (156)	NS	no
20. Elementary Lne. (‘11)	119° 15′ 16.1′′ W, 35° 25′ 20.5′′ N	Panoche clay loam (211)	GS	no
21. Beech Str. (‘11)	119° 15′ 43.5′′ W, 35° 26′ 39.6′′ N	Garces silt loam (156)	GS	yes
**Wasco**				
22. Gun Club Rd.(‘11)	119° 29′ 54.0′′ W, 35° 39′ 34.9′′ N	Garces silt loam (156)	NS	yes
23. McCoy Rd. (‘11)	119° 31′ 34.3′′ W, 35° 37′ 24.8′′ N	Garces silt loam (156)	NS	yes
**Arvin**				
24. Di Giorgio Rd. (‘11)	118° 57′ 28.7′′ W, 35° 15′ 06.6′′ N	Garces loam (180)	GS	yes
25. Bear Mt. Rd. (‘11)	118° 57′ 05.9′′ W, 35° 12′ 30.0′′ N	Garces loam (180)	GS	yes

Growth sites (GS), accumulation sites (AS) and negative sites (NS) were determined by multiplex PCR results, nd: not determined.

* Proof of rodent activity was observed in the immediate neighborhood of the sampling site. Soil disturbing activity was also observed by burrowing owls, coyotes, kit foxes, spiders or large ants at some locations. The dominant rodents observed were ground squirrels, kangaroo rats and hares.

### DNA extraction and multiplex Polymerase Chain Reaction (PCR)

DNA was extracted from well-mixed soil samples (two replicates) using the MoBio PowerSoil DNA Isolation Kit (MoBio Laboratories, Solana Beach, CA) following the manufacturer's protocol. The multiplex PCR approach developed by Greene et al. [47] and optimized for the detection of *C. immitis* from soil DNA by Lauer et al. [32] was used to determine the presence of fungi in general and specifically *C. immitis* in all soil samples with two primer pairs. Primer pair ITSC1A/ITS C2 (18S ribosomal intertranscribed spacer [ITS] region, 223 bp), which is specific for *C. immitis*, was used in combination with primer pair RDS478/RDS482 (18S ribosomal gene, 650 bp) which amplifies 18S rDNA from all fungi. The ITS region was chosen due to its high nucleotide variability. Amplified ITS fragments were extracted from the 2% Agarose Gel, extracted with the Zymo Clean Gel DNA Recovery kit (ZymoResearch, Irvine, CA), and subsequently sequenced to confirm the presence of *C. immitis*. Extracted DNA from a *C. immitis* isolate (M39), obtained from the Laboratory of Medical Mycology at the Universidad Nacional Autónoma de México was used as positive control. Negative controls and positive controls were included in all PCR's to detect contamination and to verify the amplification of a PCR product of the desired size.

Sites were *C. immitis* was detected at least twice in a deeper soil layer during the late winter/spring (February–May) when the soil is moist and the soil temperature increased were referred to as ‘growth sites’ of the pathogen in this study, assuming that the soil parameters likely supported the growth of the fungus and thus, the pathogen could be detected consecutively over a several year period in the same location, over several growth seasons. Based on the definition provided by Fisher et al. [31], growth sites are sites where physical, chemical, and biological conditions are suitable for completion of the entire growth cycle required by the organism. Thus, it could be assumed that if the pathogen finds supportive environmental conditions, it would likely expand into deeper soil layers, and not just remain on the surface which can be more hostile due to desiccation and increased uv-radiation. In fact, the majority of the soil samples that contained the pathogen in deeper soil layers also contained the pathogen in surface layers. To the contrary, sites were termed ‘accumulation sites’ in this study when the pathogen could only be detected occasionally on the surface of the sampling site and never in a deeper soil layer over a several year period. This made it likely that arthroconidia had been transported to this location by the wind, but the pathogen was never able to complete its life cycle because of non-supportive environmental conditions. ‘Accumulation sites’ were also never positive in consecutive years in contrast to ‘growth sites’. Fisher et al. [31] defined ‘accumulation sites’ as sites where arthroconidia of *Coccidioides* may have been deposited on or near the soil surface after being transported from growth sites by wind, water, organisms, or anthropogenic means. We are aware that we did not investigate the activity of the pathogen in the soil or verify its growth, and that finding the pathogen in the surface layer of the soil does not mean that it cannot grow there at all. Therefore, we have to consider that some of our results might have been false negatives.

## Results

### Remote Sensing Approach

**Figure 1:**
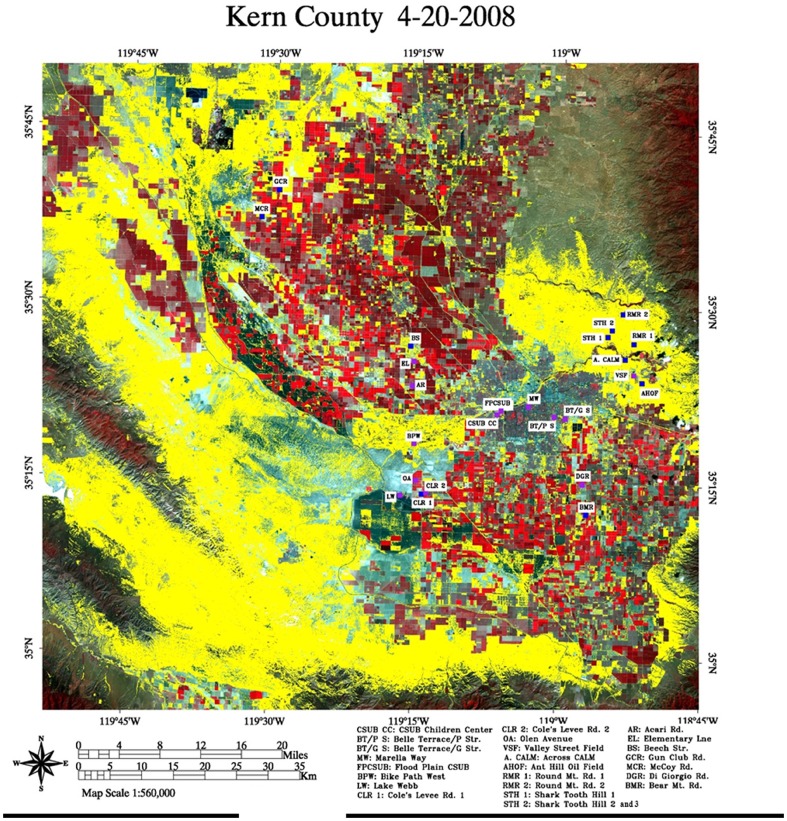
False color image of the ROI on April 20, 2008. Yellow pixels indicate locations in the STH-vegetation class, 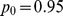 and 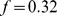.

A false color map of our ROI for April 28, 2008 was generated and is presented in figure 1, with indication of all sampling sites. Sites which were similar in vegetation to site STH, a confirmed growth site of *C. immitis*, were indicated in yellow, whereas sites that are characterized by different vegetation types appeared in various shades of green and red (agricultural fields, housing developments with gardens, higher elevated mountain slopes etc.). Results by Landsat imagery indicated large areas west of Bakersfield as potential growth sites of the pathogen, in addition to the STH area east of Bakersfield. The city of Taft southwest of Bakersfield was completely surrounded by vegetation that is similar to the vegetation type that characterizes STH. Landsat imagery furthermore indicated small pockets of potential growth sites of *C. immitis* scattered throughout the Southern San Joaquin Valley and around and within the city of Bakersfield. Overall, the yellow colors indicate that about 15% of the landscape visible in the satellite image was covered with vegetation that has the same reflection pattern as the STH vegetation.

**Figure 2:**
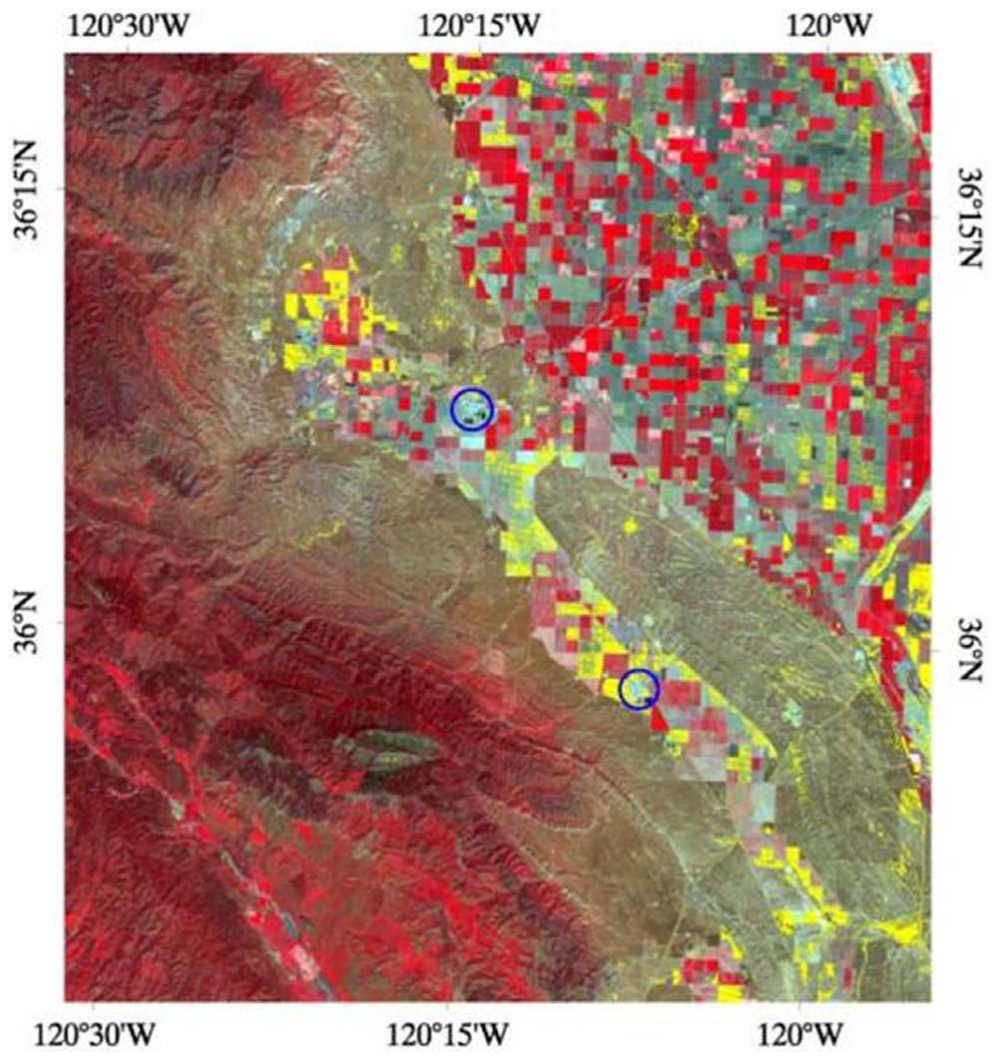
False color image of two San Joaquin Valley prisons. STH-vegetation class pixels are shown in yellow. The circles indicate the location of the prisons. Upper left: Pleasant Valley State Prison in Fresno County, California. Lower right: Avenal State Prison in Kings County, California. Images were taken on April 20, 2008. Maximum Likelihood Classification scheme was used with 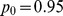.

We also applied Landsat imagery to an area northwest of Bakersfield where two prisons are located near the cities of Avenal and Coalinga (Kings County and Fresno County) (Fig. 2). In this area, the incidence of coccidioidomycosis has been observed to be large among prison inmates, so one might hypothesize that *C. immitis* could be present in the neighboring environments. And indeed, yellow areas in the immediate neighborhood of the prisons, as presented by Landsat imagery, indicated the presence of potential growth sites of the pathogen.

**Figure 3:**
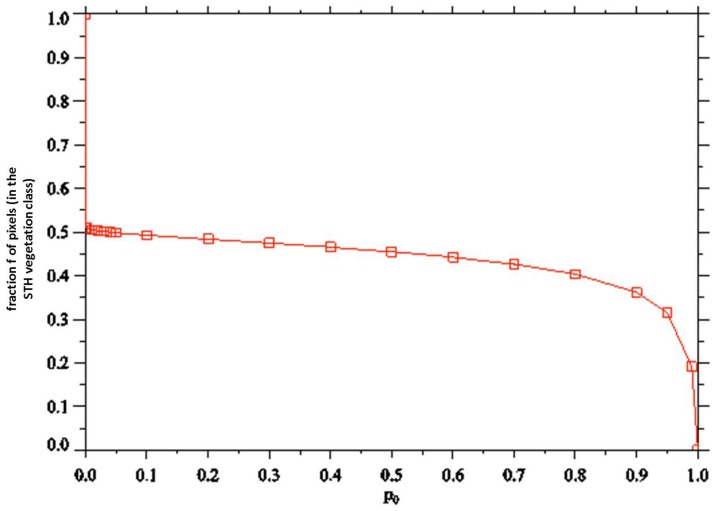
Plot of the fraction *f* of pixels in the STH-vegetation class vs. the threshold value *p_0_*. Pixels whose probability of being in the STH-vegetation class is 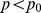 are left unclassified. Pixels with 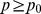 are put in the class. In this plot, 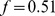 for 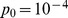, and 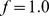 for 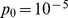.

Overall, 4 false color maps were generated for our ROI showing results for April 2008 to April 2011 (one for each April, see Table 2). For each satellite picture we obtained one STH vegetation profile. Therefore, it did not matter if variation in the vegetation occurred. The April profiles were considered the most definitive for our work, because in early spring, the climate (soil and air temperature and humidity) still support the growth of the vegetation, and grasses and herbs which are characteristic for the STH- vegetation profile have not dried up yet, compared to the summer months. A validation of our approach is shown in figure 3. In this figure, the corresponding plot of ƒ vs. *p_0_* is presented. This figure also presents the dependence of the fraction ƒ on *p_0_*. It can be pointed out that when *p_0_* drops from 1, ƒ changes from zero to 0.36 at 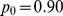, but then increases slowly with decreasing *p_0_* until 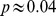, where 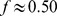, and jumps to 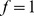 for 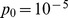. Most likely, this is due to the STH-vegetation class being quite distinct from all other possible spectral classes in the ROI, with a large distance (in pixel space) from those classes. Otherwise, discrete increases in ƒ with decreasing *p_0_* would be expected as other surface types get merged into the STH-vegetation class.

**Table 2:** Probability that the sites fall in the STH-vegetation class, as predicted by Landsat data.

sampling sites and year sampled	*p_0_* = 0.90				*C. immitis* growth site (multiplex PCR)	*p_0_* = 0.95			
**Bakersfield city**	4/20/08	4/23/09	4/26/10	4/29/11		4/20/08	4/23/09	4/26/10	4/29/11
1. CSUB Children Center (′08, ′09)	N	N	N	N	accumulation site*	N	N	N	N
2. Belle Terrace/P Str. (‘11)	**Y**	N	N	N	growth site*	N	N	N	N
3. Belle Terrace/Gay Str. (‘11)	N	N	**Y**	N	growth site	N	N	**Y**	N
4. Marella Way (‘11)	N	N	N	N	negative site*	N	N	N	N
5. Flood Plain CSUB (′08, ′09)	N	N	N	N	accumulation site	N	N	N	N
**SW Bakersfield**									
6. Bike Path West (′08, ′09)	N	N	N	N	negative site	N	N	N	N
7. Lake Webb (′08, ′09)	N	N	N	N	accumulation site	N	N	N	N
8. Cole's Levee Rd. I (′08, ′09, ‘11)	**Y**	**Y**	**Y**	**Y**	growth site	**Y**	N	N	N
9. Cole's Levee Rd. II (‘11)	**Y**	**Y**	**Y**	**Y**	growth site	**Y**	**Y**	**Y**	N
10. Olen Avenue (‘11)	N	N	N	N	growth site	N	N	N	N
11. Valley Street Field (′08, ′09)	N	N	N	N	accumulation site	N	N	N	N
**NE Bakersfield**									
12. Across CALM (‘11)	**Y**	**Y**	N	N	growth site	**Y**	**Y**	N	N
13. Ant Hill Oil Field (′08, ′09, ′11)	**Y**	**Y**	**Y**	N	growth site	**Y**	**Y**	**Y**	N
14. Round Mt. Rd. I (′08, ′09)	**Y**	**Y**	**Y**	N	accumulation site	**Y**	**Y**	**Y**	N
15. Round Mt. Rd. II (′08, ′09)	**Y**	**Y**	N	N	negative site	**Y**	**Y**	N	N
16. Sharktooth hill I	**Y**	**Y**	**Y**	**Y**	nd*	**Y**	**Y**	**Y**	**Y**
17. Sharktooth hill 2	**Y**	**Y**	**Y**	**Y**	nd**	**Y**	**Y**	**Y**	**Y**
18. Sharktooth hill 3 (′11)	**Y**	**Y**	**Y**	**Y**	growth site	**Y**	**Y**	**Y**	**Y**
**NW Bakersfield**									
19. Acari Rd. (‘11)	N	N	N	N	negative site	N	N	N	N
20. Elementary Lne. (‘11)	N	N	N	N	growth site	N	N	N	N
21. Beech Str. (‘11)	N	**Y**	N	**Y**	growth site	N	**Y**	N	**Y**
**Wasco**									
22. Gun Club Rd.(‘11)	**Y**	**Y**	**Y**	**Y**	negative site	**Y**	**Y**	N	**Y**
23. McCoy Rd. (‘11)	**Y**	**Y**	**Y**	**Y**	negative site	**Y**	N	**Y**	**Y**
**Arvin**									
24. Di Giorgio Rd. (‘11)	N	**Y**	N	N	growth site	N	**Y**	N	N
25. Bear Mt. Rd. (‘11)	**Y**	N	N	N	growth site	**Y**	N	N	N
**Fraction of area covered by class (%)**	35	42	24	28		32	39	22	26

(**Y** = in class [indicated in bold], N = not in class).

nd* = not determined in this study.

nd** = not determined in this study, but confirmed as growth site by Swatek (1970).

growth site* =  sites were *C. immitis* was detected at least twice in a deeper soil layer during the late winter/spring (February-May).

accumulation site* =  the pathogen could only be detected on the surface of the sampling site and never in a deeper soil layer over a several year period.

negative site* = the pathogen could not be detected in any of the soil samples using the multiplex PCR method as described in this study.

**Figure 4:**
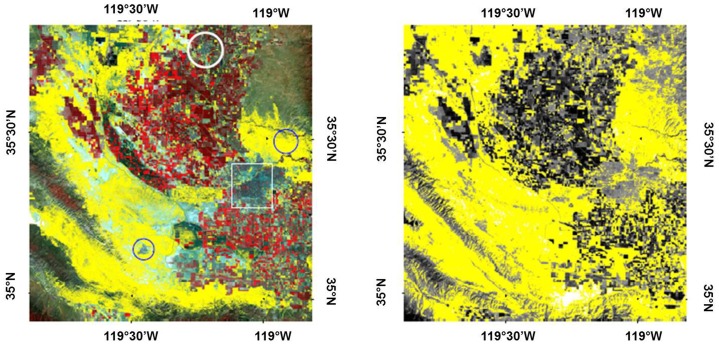
Left: False color image of the ROI on April 20, 2008. Yellow pixels indicate locations in the STH-vegetation class. 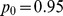 and 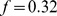. The square denotes the location of the city of Bakersfield, the circle on the top indicates the city of Delano, the circle on the right indicates the location of STH, and the circle on the left indicates the location of the city of Taft. **Right**: Spectral class comparison. STH-thermal class is shown in yellow for the same April 20, 2008 image. Parallelepiped scheme was used with thresholds 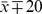, with 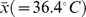 the average surface temperature on the STH training pixels, and 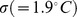 the corresponding standard deviation.

As a further consistency check, we also wanted to examine the extent to which the STH-vegetation and STH-thermal classes overlapped and if growth sites of the pathogen could be predicted by soil thermal data (tables 2 and 3, figure 4). Thus, we obtained the STH-thermal class by implementing a parallelepiped scheme as described before (see methods). For 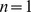, only sites 13, 14, 16 and 21 were included in the STH-thermal class (April 2008, data not shown). Sites 8, 9, 12–18, 21, and 22–24 were added when 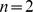. Site 11 was included when 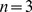. Site 25 (one of the strongest growth sites of the pathogen) was never included. Figure 4 (right) shows our results for 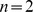. We present this figure here because site 8, which we identified as a strong growth site of the pathogen, came into the STH-thermal class for this value of *n* (but site 8 was not in the class for 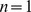). By evaluating the agreement between satellite imagery (STH-vegetation class and STH-thermal class, data from 4 consecutive years), we found that both data sets disagreed in 12% (sites 6, 11 and 25). Almost all sites that fell into the STH-vegetation class also fell into the STH-thermal class (see tables 2 and 3).

**Table 3:** Probability that the sites fall in the STH-thermal class, as predicted by Landsat data.

sampling sites and year sampled	n = 3				*C. immitis* growth site (multiplex PCR)	n = 2			
**Bakersfield city**	4/20/08	4/23/09	4/26/10	4/29/11		4/20/08	4/23/09	4/26/10	4/29/11
1. CSUB Children Center (′08, ′09)	N	N	N	N	accumulation site	N	N	N	N
2. Belle Terrace/P Str. (‘11)	N	N	**Y**	**Y**	growth site	N	N	N	**Y**
3. Belle Terrace/Gay Str. (‘11)	N	**Y**	**Y**	N	growth site	N	**Y**	**Y**	N
4. Marella Way (‘11)	N	N	N	N	negative site	N	N	N	N
5. Flood Plain CSUB (′08, ′09)	N	N	N	N	accumulation site	N	N	N	N
**SW Bakersfield**									
6. Bike Path West (′08, ′09)	N	**Y**	**Y**	**Y**	negative site	N	N	N	N
7. Lake Webb (′08, ′09)	N	N	N	N	accumulation site	N	N	N	N
8. Cole's Levee Rd. I (′08, ′09, ‘11)	N	**Y**	**Y**	**Y**	growth site	N	**Y**	N	**Y**
9. Cole's Levee Rd. II (‘11)	N	**Y**	**Y**	N	growth site	N	**Y**	**Y**	N
10. Olen Avenue (‘11)	N	N	N	N	growth site	N	N	N	N
11. Valley Street Field (′08, ′09)	N	N	**Y**	N	accumulation site	N	N	N	N
**NE Bakersfield**									
12. Across CALM (‘11)	**Y**	N	**Y**	N	growth site	**Y**	N	N	N
13. Ant Hill Oil Field (′08, ′09, ′11)	**Y**	**Y**	**Y**	N	growth site	**Y**	**Y**	N	N
14. Round Mt. Rd. I (′08, ′09)	**Y**	**Y**	**Y**	N	accumulation site	**Y**	**Y**	**Y**	N
15. Round Mt. Rd. II (′08, ′09)	**Y**	**Y**	**Y**	N	negative site	**Y**	**Y**	N	N
16. Sharktooth hill I	**Y**	**Y**	**Y**	**Y**	nd*	**Y**	**Y**	**Y**	**Y**
17. Sharktooth hill 2	**Y**	**Y**	**Y**	N	nd**	**Y**	**Y**	N	N
18. Sharktooth hill 3 (′11)	**Y**	**Y**	**Y**	N	growth site	**Y**	**Y**	N	N
**NW Bakersfield**									
19. Acari Rd. (‘11)	N	N	N	N	negative site	N	N	N	N
20. Elementary Lne. (‘11)	N	N	N	N	growth site	N	N	N	N
21. Beech Str. (‘11)	N	**Y**	**Y**	N	growth site	N	**Y**	**Y**	N
**Wasco**									
22. Gun Club Rd.(‘11)	N	N	**Y**	**Y**	negative site	N	N	**Y**	**Y**
23. McCoy Rd. (‘11)	**Y**	**Y**	**Y**	**Y**	negative site	**Y**	N	**Y**	**Y**
**Arvin**									
24. Di Giorgio Rd. (‘11)	**Y**	**Y**	**Y**	**Y**	growth site	N	**Y**	**Y**	**Y**
25. Bear Mt. Rd. (‘11)	N	N	N	N	growth site	N	N	N	N
**Fraction of area covered by class (%)**	35	42	24	28		32	39	22	26

(**Y** = in class [indicated in bold], N = not in class).

nd* = not determined in this study.

nd** = not determined in this study, but confirmed as growth site by Swatek (1970).

### Soil series and soil parameters

**Figure 5:**
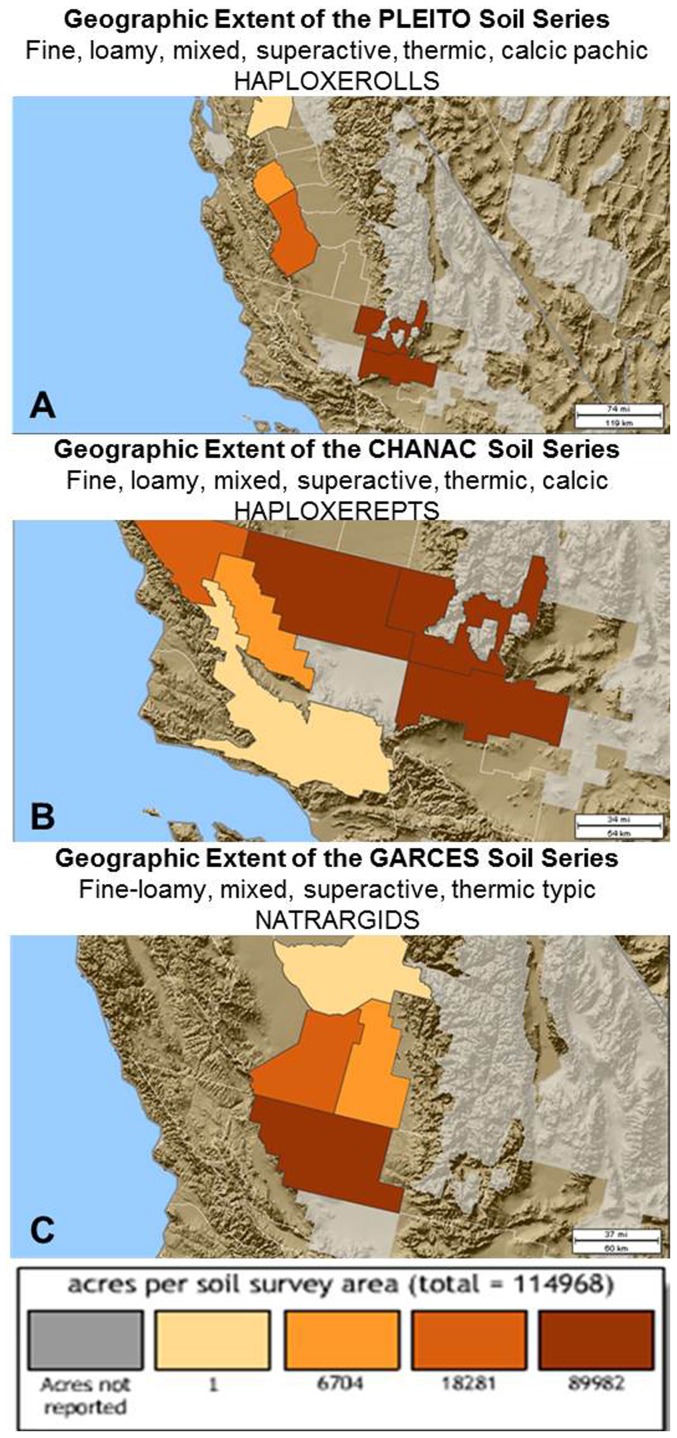
Extend of soil series in the San Joaquin Valley, CA, which can support the growth of *C. immitis*. **A**: Pleito (brown: SE and NE Kern County, dark orange: W Fresno County, light orange: W Merced County, tan: San Joaquin County) **B**: Chanac (brown: SE, NE and NW Kern County, dark orange: San Louis Obispo County [Paso Robles area], light orange: San Luis Obispo County, [Carrizo Plains]), and **C**: Garces soil series (brown: NW Kern County, dark orange: Kings County, light orange: W Tulare County, tan: E Fresno Area), Center for Environmental Informatics at Pennsylvania State University (CEI), http://www.cei.psu.edu/soiltool/semtool.html .

By using the soil series extent mapping tool, we found that the soil series and soil groups in which the pathogen was detected around Bakersfield, California, belonged to the Garces (Natragid, sites CLR, Bear Mt. Rd.), Chanac (Haploxerept, site AHOF), and Pleito (Haploxeroll, sites STH1 and 2 and 3) series. These soil series are not restricted to the Southern San Joaquin Valley. See figure 5 for a distribution of these soil series in California. Soils that belonged to the Chanac soil series can also be found in western Arizona and southern Nevada. All soils were of mixed mineralogy, had a superactive cation exchange capacity, and were thermic soils with predominantly fine loamy particles. Soils that belonged to these soil series are among the dominant soils in the Southern San Joaquin Valley, especially Kern County and Kings County, but can also be found in northern and western California. The use of software such as the USDA websoilsurvey database, as well as tools available at the Center of Environmental Informatics (CEI) have been found to be very valuable in obtaining information about physical and chemical parameters of soils that could support the growth of *C. immitis*. Detailed information about soil type, landform, dominant parent material of the soil, as well as soil physical and chemical parameters are listed for all sampling sites in table 4. Using these tools, information about land use, vegetation, mean annual soil temperatures, and geographic setting was accessed as well and is summarized in table S1 in file S1.

**Table 4:** Detailed physical and chemical information obtained from the USDA websoilsurvey database for all sites included in this study.

soil	sampling sites					
parameters soil parameters	Elementary Lne.	across CALM Ant Hill Oil Field	Bear Mt. Rd. Di Georgio Rd. Olen Ave. Cole's Levee Rd.	Sharktooth hill	Belle Terrace/Gay Str. Belle Terrace/P Str. Marella Way	McCoy Rd. Gun Club Rd. Acari Rd. Beech Str.
**soil type**	Panoche clay loam	Chanac clay loam	Garces loam	Pleito-Trigo-Chanac complex	Kimberlina-Urban land Cajon complex	Garces silt loam
**landform**	alluvial fans	fan remnants	Alluvium derived from granitoid	Fan remnants, stream terraces	alluvial fans	rims on basin floors
**parent material**	alluvium derived from igneous and sedimentary rock	alluvium derived from mixed	alluvium derived from granitoid	Alluvium derived from mixed	alluvium derived from igneous and sedimentary rock	alluvium derived from granite
(map unit symbols)	211	130/131	180	205	180	156
**Physical parameters**						
Surface texture	clay loam	clay loam	clay loam	clay loam	loamy sand	silt loam
*% clay*	31	31	25.5	30	12	26.8
*% sand*	35.4	35.4	38	33.5	71.3	34.2
*% silt*	33.6	33.6	36.5	36.5	16.7	39.1
*Available water capacity (cm/cm)*	0.17	0.17	0.21	0.16	0.12	0.11
*Available water supply (0–25 cm)*	4.25	4.25	5.04	3.69	2.64	2.7
Organic matter	0.25	0.75	0.98	1.5	0.75	0.06
Water content (15 bar)	18.9	18.2	16.7	17.2	8.7	16.2
*Water content (1/3 bar)*	32	30.1	30.9	27.8	17.7	30.2
Sat. hydraulic conductivity (Ksat) (micrometers/s)	9	9	8.37	2.82	28	0.8362
**Chemical parameters**						
*pH*	7.9	7.9	8.5	7.8	7.5	8.9
CaCO3	3	3	3	0	3	3
Cation Exchange Capacity (CEC7)	15	24.4	20.6	24.3	7.5	13.1
Gypsum	0	0	0	0	0	0
Sodium adsorption ratio (SAR)	0	0	2	0	0	14
Electrical conductivity (EC)	1	0	5	0.5	1	10.2

Indicated in cursive are the parameters which seemed to be most important to distinguish *C. immitis* growth sites from negative sites.

### Detection of *C. immitis* by multiplex PCR

**Figure 6:**
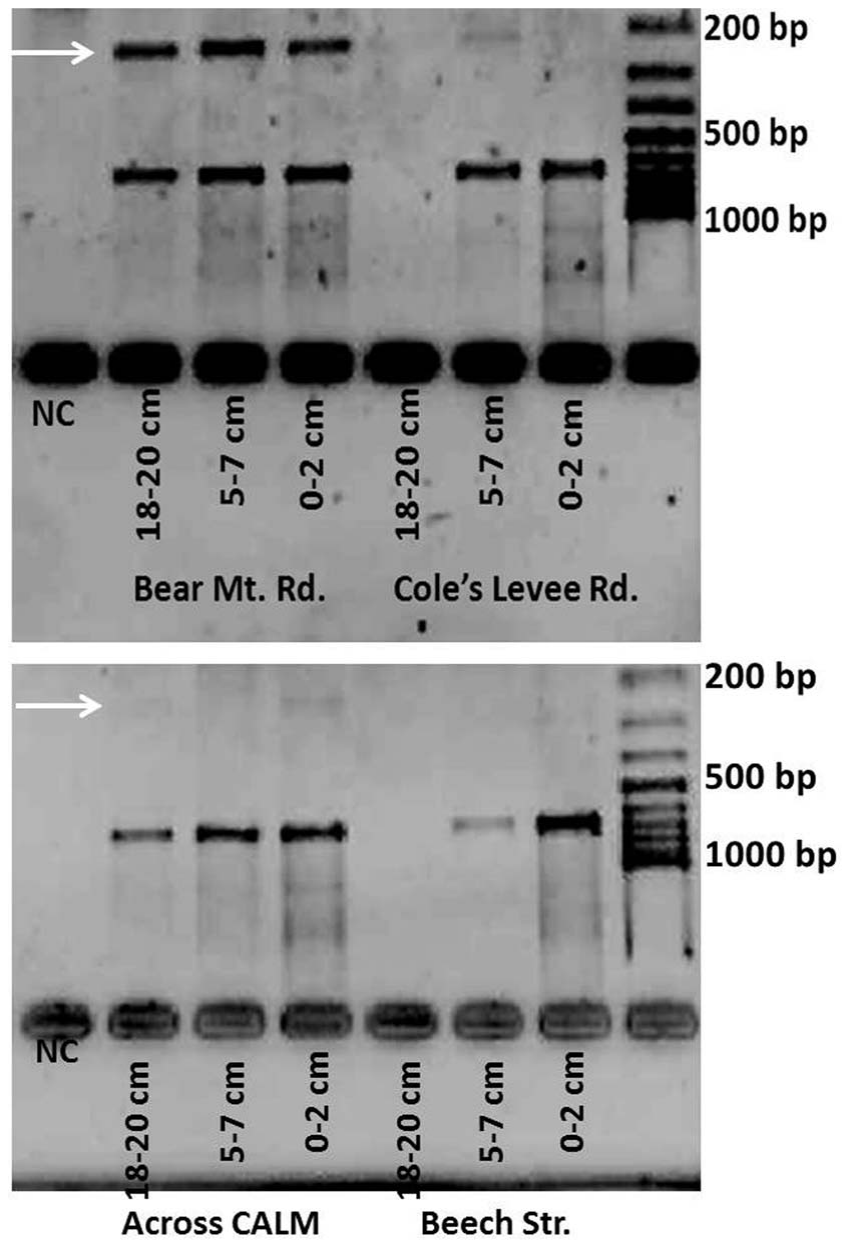
Example of multiplex PCR results. White arrows point on a 223 bp fragment that represents *C. immitis*. Site Bear Mt. Rd. shows the strongest ITS amplicons in all soil layers, whereas sites Cole's Levee Rd. and site Across CALM gave a weaker signal in some soil layers, and site Beech Str. was negative. NC  =  negative control. Bands that indicate the presence of the pathogen in the 2% Agarose gel were confirmed to origin from *C. immitis* by sequencing.

In addition to the two growth sites of the pathogen that were detected in 2008 and 2009 (Cole's Levee Rd. [CLR], Ant Hill Oil Field [AHOF], see [32]), we were able to detect additional “hot spots” of the pathogen in 2011. These sites were located within Bakersfield city, in the southwest, northeast and northwest of Bakersfield, and near Arvin, California (see table 1). Sites where the pathogen was detected more than once in a deeper soil layer were considered growth sites, whereas sites where the pathogen was detected occasionally in the surface layer only were considered accumulation sites. Sites were *C. immitis* was never detected were considered negative sites and included areas within Bakersfield city, an area northwest of Bakersfield, and 2 sites near Wasco, California. Fungal DNA could be detected in all soil samples. Of all sites investigated in 2011 (two growth sites investigated in 2008/09 [CLR and AHOF], and 14 new sites, out of 16 sites altogether for 2011), 4 sites (25%) were found negative, and 12 sites (75%) were confirmed as growth sites of the pathogen. No new accumulation sites were discovered in 2011. The site at Bear Mt. Road was the strongest growth site of *C. immitis* for the 2011 sampling set (positive for *C. immitis* from Jan–Apr). For an example of multiplex PCR results, see figure 6.

### Correlation between Landsat imagery and multiplex PCR results

By comparing results obtained by satellite imagery (STH-vegetation class) and multiplex PCR, we found that in ∼74% (17 out of 23 tested sites) the satellite imagery results and the results obtained by multiplex PCR agreed at least in one year out of four years 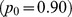. When 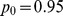, the agreement was ∼70% (16 sites). When satellite imagery based on STH-thermal class was compared with multiplex PCR results, we found that both methods agreed only in 61% 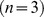 or 65% 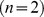 (table 5). Tables 2 and 3 show the probability that the sites fall in the ‘*C. immitis* growth area’ based on Landsat data in comparison to results obtained by multiplex PCR. We set Red, Green and Blue (RGB) to TM bands 4, 3, and 2 respectively. With this choice, the different depths of red indicated different plant associations. These maps were the result of implementing the MLC method with 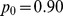
and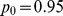. Two of the three sites at STH listed in table 1 were confirmed growth sites of *C. immitis*. STH site 2 was confirmed as a growth site by Frank Swatek (Fisher FS, personal communication based on [30]), and STH site 3 was confirmed as a growth site by multiplex PCR in this study. Sampling site 8 (Cole's Levee Rd. I), which was determined as a strong growth site of the pathogen by multiplex PCR in every year, became included in the ‘*C. immitis* growth area’ based on Landsat data when *p_0_* was reduced to 0.95. Sampling site 7 (Lake Webb, accumulation site, located less than 1 mile west of CLR) was added when *p_0_* was reduced to 0.10. Sampling sites 1, 5, 6, and 11, (near Children Center, Flood Plain, Bike Path West, and Valley Street Field) never got added for 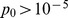. This was consistent with results obtained by multiplex which confirmed the absence of the pathogen (site 6, negative site), or which detected the pathogen occasionally in surface samples only (sites 1, 5 and 11). Sites, 1, 5 and 11 were termed accumulation sites, where the arthroconidia had been likely transported to by the wind, and where the presence of the pathogen could not be detected in deeper layers by multiplex PCR. We interpret this to mean that STH was quite representative of the *C. immitis* ecological niche within our ROI. However, in some occasions the prediction made by satellite imagery to indicate soils that could potentially harbor the pathogen could not be confirmed by multiplex PCR. Of all 25 sites included in this study, only sites 8 and 9 (Cole's Levee Rd. I and II) fell in the STH-vegetation class in each year when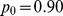. These sites were confirmed as positive for *C. immitis* by multiplex PCR. Other sites that were confirmed as growth sites of the fungus by our culture independent approach fell in this class at least on one occasion out of four when 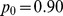
(sites 2, 3 [Belle Terrace/P Str. and Belle Terrace/Gay Str.], 12 [Across CALM], 13 [AHOF], 21 [Beech Str.], 24 [Di Giorgio Rd.], and 25 [Bear Mt. Rd.]). These sites were still included in the STH-vegetation site when 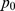 was increased to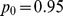, with the exception of site 25. Of all sites that were found to be *C. immitis* growth sites by multiplex PCR, two sites were never indicated as a potential growth site by the MLC method (site 10 [Olen Ave.] and site 20 [Elementary Lne.]), but the pathogen was present in soil samples from both sites as confirmed by multiplex PCR (see Discussion). Furthermore, two sites that were indicated as potential growth sites of the pathogen by Landsat imagery at all times when 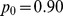, could not be confirmed by multiplex PCR to harbor the pathogen. These sites were located near Wasco, California (NW of Bakersfield), (sites 22 [Gun Club Rd.] and 23 [McCoy Rd.]).

**Table 5:** Agreement between multiplex PCR and MLC for the STH vegetation class and the STH-thermal class to predict growth sites of *C. immitis* (to agree a prediction by either multiplex PCR or MLC must be confirmed at least once for the four years by the other method).

	STH-vegetation class	
	*p_0_* = 0.90	*p_0_* = 0.95
**multiplex PCR and MLC agree**	17 (74%)	16 (70%)
**multiplex PCR predicts growth site and MLC disagrees**	2 (9%)	3 (13%)
**MLC predicts growth site and multiplex PCR disagrees**	4 (17%)	4 (17%)

	STH-thermal class	
	n = 3	n = 2
**multiplex PCR and MLC agree**	14 (61%)	15 (65%)
**multiplex PCR predicts growth site and MLC disagrees**	3 (13%)	4 (17.5%)
**MLC predicts growth site and multiplex PCR disagrees**	6 (26%)	4 (17.5%)

From altogether 25 sites, only 23 were considered, because no multiplex PCR results were obtained for STH sites I and II.

Changes in extend of areas (km^2^) that fell into the STH-vegetation class were observed for the sampling period until early 2014 and are displayed in table S2 in file S1. The year with the highest precipitation (2011) had the lowest area of vegetation that belonged into the STH-vegetation class in comparison to the years 2008 and 2009 which were characterized by a significantly reduced amount of precipitation and showed an increased area of vegetation that belonged into the STH-vegetation class (see figure S1).

## Discussion

The purpose of our study was to identify soil types in Kern County that could support the growth of *C. immitis* by combining Landsat imagery (based on vegetation and soil temperature), and soil parameter information (from 25 sites) with a culture independent PCR-based method to detect the pathogen. We showed that satellite imagery, combined with soil parameter information, can provide a map of locations within our ROI, where *C. immitis* might reasonably be expected to be found. We were able to verify the presence of the pathogen by a multiplex PCR method in about 74%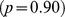, when soil samples were investigated over a 4 year period. However, for about a quarter of our sites (26%), results obtained by Landsat imagery and multiplex PCR did not correlate. The reasons for this observation could be multifold. Some main factors to be considered are: 1) The amount of *Coccidioides* DNA extracted from the soil might have been under the detection limit of our PCR based methods (sites 22 and 23), or 2) the resolution of the satellite imagery might not have been detailed enough (site 10, a small site of only 10 m^2^), and 3) the distribution of *Coccidioides* in the soil might have been spotty, and the positive site was missed (sites that were not positive for the pathogen in all sampling years). A closer look at sites where satellite imagery and soil parameter data indicated potential growth sites for *C. immitis* also revealed that these sites were not uniform in regard to plant coverage, distribution, and diversity, thus, generating microhabitats for soil microorganisms that most likely would be quiet distinct, especially in and around the rhizosphere [32]. Other factors, such as fluctuation in climate and pollution of the soil might have had an impact on our analyses as well. Furthermore, it has to be considered that *C. immitis* might be able to persist in soils that have been converted to agricultural fields for an unknown amount of time, but its arthroconidia might never germinate and grow into vegetative hyphae. These sites could be termed dormant sites (e.g. site 20, an orchard with young almond trees). To assess these impacts on our results was not the focus of our work, but we are aware of these limitations. In previous research we have investigated the limitation of the multiplex PCR approach to detect *C. immitis*, (see [33] for results of primer efficiency). Briefly, we found that the sensitivity of the diagnostic PCR (ITS primer pair) was reduced compared to the primer pair that amplifies 18S rDNA fragments of all fungi (RDS primer pair).

In previous work [23]–[25], it was suggested that some environmental fluctuations are a fundamental link missing from coccidioidomycosis incidence statistical modeling schemes. One important aspect to investigate is whether fluctuations in the STH-vegetation class can provide this connection, and be statistically linked to the observed variations in incidence of valley fever. In this regard, one effect to consider is the extent to which this RS approach continues to be valid through the seasons. In the spring, when plants are blooming, the different vegetation types have different spectral signatures. As the weather dries and plants wither, the spectral signatures of the relevant vegetation types may become less distinct. Thus, the vegetation on STH may not be as good a marker for *C. immitis* in the fall, as it is in the spring. The implicit assumption in this study is that the STH environment is the only type of environment which harbors *C. immitis* within the San Joaquin Valley area of Kern County. We presented in this paper arguments to support this assumption; nevertheless, it would be useful to find more similarly suitable test sites to further corroborate our findings, or to find slightly different ecotypes that support *C. immitis*, beside of those detected in this study.

We also observed changes in the extent of the STH-vegetation class over time. A comparative analysis of precipitation between 2008 and 2011 (up to early 2014) suggest that years with a reduced precipitation (drought) favor plants of the STH-vegetation class, but other factors likely play a role as well, such as development and changes in land use (see figure S1 and table S1 in File S1), which was not assessed in this study.

Compared to the STH-vegetation class data, the STH-thermal data showed considerably more variation for the four different years, as expected. The vegetation on a certain day in each year may be very similar, but soil temperatures might be more variable in different years (data for 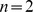 and 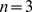 can be seen in table 3, no data is shown for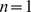). Other strong growth sites of the pathogen (sites 8, 9, and 13) were also not consistently included in all years, not even with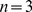. It should also be noted that sites 6 (negative site) and 11 (accumulation site) were never included into the STH-vegetation class by satellite imagery, but were included in the STH-thermal class when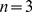. We concluded therefore, that the STH-thermal classes alone might not be sensitive enough to predict growth sites of *C. immits*. Site 25 was never included (thermal class) maybe because of the limited resolution of the satellite imagery, as discussed earlier. It is important to keep in mind that the TM-6 image tells us surface temperatures. It may very well be that what matters is the temperature below the surface [31]. For our ROI, all landcovers were similar on the macroscale; therefore, apparent temperatures were appropriate for comparison purposes.

To improve the value of satellite imagery data, the actual spectral reflectance profiles of various soil components could be included to complement the satellite data (for details see http://www.africasoils.net/data/ldsf-description) in future studies. A time series analysis could also be considered, if feasible. Phenology development throughout the year can make the analysis more specific to a particular vegetation type. Niche modeling rather than automated classification could be considered as well to obtain a richer output that indicates variables of importance. However, a large dataset would be necessary that would include presence and also absence data of the pathogen in a certain type of soil at a certain time with presence or absence of a certain type of vegetation. Furthermore, it should be considered that broad band signatures over larger geographic areas and ecotones might not be precise enough to be useful in predicting growth sites of a pathogen, especially when the pathogen could be adapted to grow in a variety of different ecosystems.

Our results indicated that strong growth sites of the pathogen were likely associated with 3 different USDA soil map units (180, 131, and 205), which were all loamy sands. Several sites around Bakersfield, California, that fell into one of these map units were indeed growth sites of *C. immitis*, as confirmed by multiplex PCR, and were similar in vegetation compared to the STH area. These types of soils are not restricted to the Southern San Joaquin Valley, but can be found in other areas of California as well. One could hypothesize that with a drier and warmer climate, as it is predicted for California in the near future [51], [52], *C. immitis* might be able to expand its current range. In our study, we focused on soil samples from only one County, the above mentioned Kern County in the Southern San Joaquin Valley of California, a highly endemic area for *C. immitis*. The soil types investigated here did not comprise all types that can be found in our ROI. Even though Kern County is a hot spot of *C. immitis* with the highest incidence of coccidioidomycosis documented for as long as incidence data is recorded in California, we cannot conclude that soils that predominate in this area are the ones that also predominantly support the growth of the pathogen. A more rigorous sampling framework should be attempted in the future that would include locations beyond Kern County covering as wide a range of habitats as possible to correctly determine growth sites of *C. immitis*, as well as determining sites that are not supporting the growth of the pathogen. Developing such a sampling plan should include stratification, replicate sampling, and determination of important chemical and physical soil parameters, including investigations in other countries where coccidioidomycosis occurs would be of value as well. The ultimate goal would then be to generate a U.S. or America-wide database of occurrence and absence of *Coccidioides* spp. Such a database could be useful for characterizing the ecological niche for both *Coccidioides* species, and could indicate a variety of supporting ecosystems, as well as being an advisory public health tool, to reduce incidence of coccidioidomycosis in Kern County and elsewhere.

In conclusion, the combination of the methods used in our research can be used to generate maps that indicate potential growth sites of *C. immitis*, and thus serve as a tool to further investigate the ecological niche occupied by the pathogen in the Southern San Joaquin Valley and beyond in more detail. Recent advances in computer processing and geographic information system and global positioning system technologies facilitate integration of remote sensing data, such as environmental parameters with disease incidence data, so that models for disease surveillance and control can be developed [53], [54].

## Supporting Information

Figure S1 **Cumulative monthly precipitation (inches) over time for the Southern San Joaquin Valley, assembled from 5 stations (Calaveras Big Trees [CVT], Hetch Hetchy [HTH], Yosemite HQ [YSV], North Fork RS (NFR), and Huntington Lake (HNT]) obtained from the California Data Exchange Center at http://cdec.water.ca.gov/snow_rain.html).**
(TIF)

File S1 **Supporting tables. Table S1.** Detailed soil series descriptions of sites which were found to be growth sites of *C. immitis*. **Table S2.** Extend of STH-vegetation class in our ROI between 2008 and 2011 based on satellite imagery.
(DOCX)
