# Supplementary figures and images for: Combining Forces - The Use of Landsat TM Satellite Imagery, Soil Parameter Information, and Multiplex PCR to Detect Coccidioides immitis Growth Sites in Kern County, California
Source: PLoS One. 2014 Nov 7;9(11):e111921. doi: 10.1371/journal.pone.0111921 (PMC4224400; doi:10.1371/journal.pone.0111921)

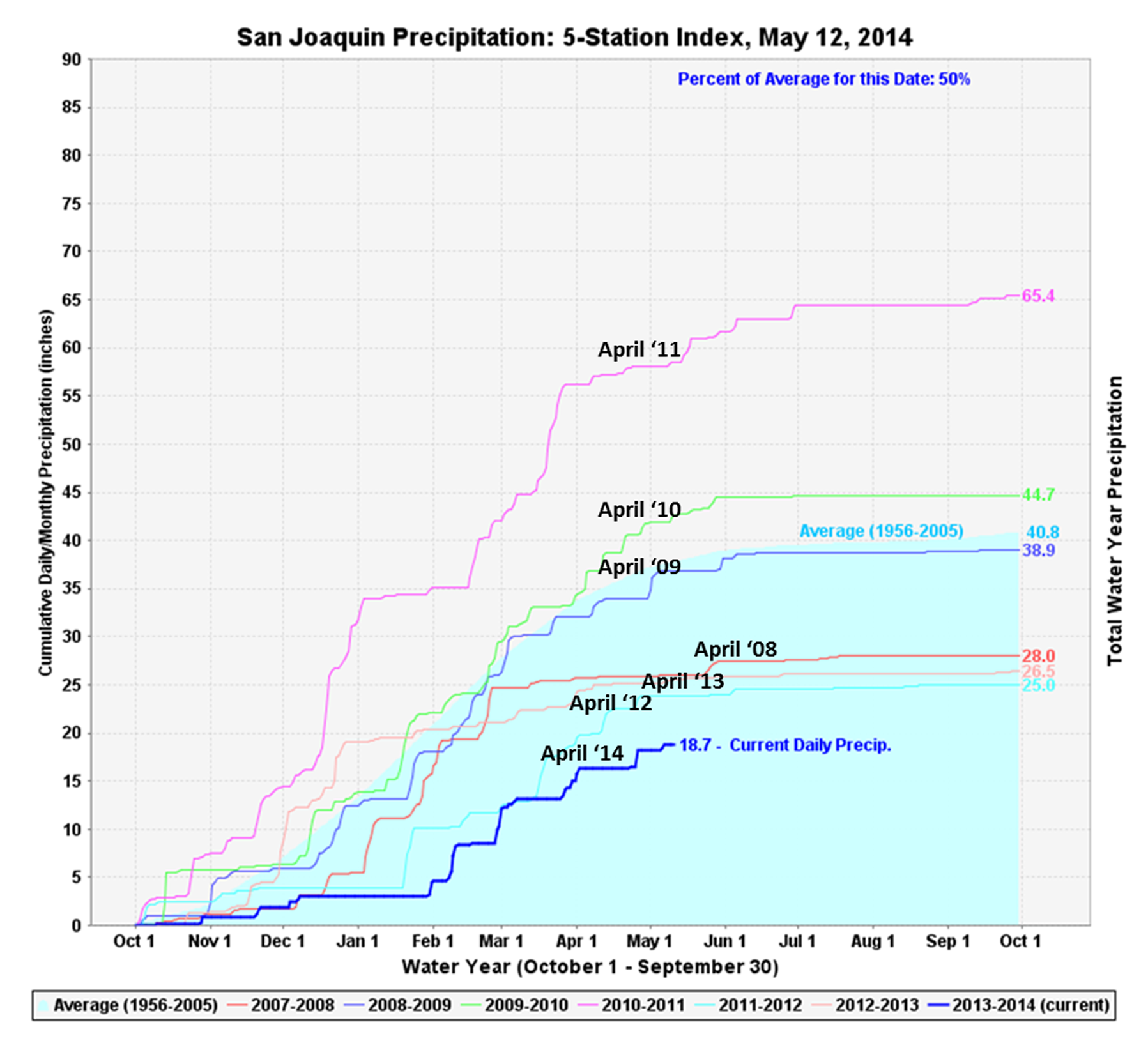

Supplement: Figure S1 — Cumulative monthly precipitation (inches) over time for the Southern San Joaquin Valley, assembled from 5 stations (Calaveras Big Trees [CVT], Hetch Hetchy [HTH], Yosemite HQ [YSV], North Fork RS (NFR), and Huntington Lake (HNT]) obtained from the California Data Exchange Center at http://cdec.water.ca.gov/snow_rain.html). (TIF) [file pone.0111921.s001.tif]
