# Supplementary material for: Combining Forces - The Use of Landsat TM Satellite Imagery, Soil Parameter Information, and Multiplex PCR to Detect Coccidioides immitis Growth Sites in Kern County, California
Source: PLoS One. 2014 Nov 7;9(11):e111921. doi: 10.1371/journal.pone.0111921 (PMC4224400; doi:10.1371/journal.pone.0111921)
Supplement: File S1 — Supporting tables. Table S1. Detailed soil series descriptions of sites which were found to be growth sites of C. immitis. Table S2. Extend of STH-vegetation class in our ROI between 2008 and 2011 based on satellite imagery. (DOCX) [file pone.0111921.s002.docx]

**Supplementary Material**

| **Table S1.** Detailed soil series descriptions of sites which were found to be growth sites of *C. immitis*. | |
| --- | --- |
| **Garces** | **Soil Series Location and Description** |
| Soil survey area | Kern County (NW part), Kings County, Tulare County (Western part), Eastern Fresno Area |
| Sites investigated in this study | Cole's Levee Rd. Olen Ave, Di Georgio Rd, and Bear Mountain Road (map soil unit 180) |
| Taxonomic class | fine-loamy, mixed, superactive, thermic Typic Natragids |
| Typical Pedon | silt loam |
| vegetation | red brome, *Atriplex* spp., filaree, scattered saltbush, annual grasses and forbs |
| Type Location | Kern County, CA, West of Wasco, Lost Hills NorthEast Quadrangle |
| Geographic setting | alluvial fans, terraces, and basin rims (granite rock sources) |
| Latitude/Longitude | 35 º, 38’, 17” North, 119 º, 31’, 50’ West |
| soil temperature | always above 8 ºC, mean annual 17.8-20 ºC |
| mean annual precipitation | 127-203 mm |
| **A horizon** parameter** |  |
| soil reaction | moderate alkaline, pH 8.2 |
| water content | soil between the depths of 10.2-30.5 cm is dry from April to January |
| organic matter | less than 0.5 % |
| clay percentage | 10-18% |
| Sodium Adsorption Ratio | 1-25 |
| Electrical conductivity | 2-8 decisiemens |
| **Pleito** |  |
| Soil survey area | Kern County (NE part and SW part), Fresno County (W part), Merced County (W part), San Joaquin County |
| Sites investigated in this study | Sharktooth Hill (STH1-3, map soil units 205 and 305) |
| Taxonomic class | fine-loamy, mixed, superactive, thermic Calcic Pachic Haploxerolls |
| Typical Pedon | sandy clay loam, used for livestock grazing |
| vegetation | wild oats, ripgut brome, red brome, soft chess, other annual grasses, scattered perennial grasses and forbs |
| Type Location | Kern County, El Tejon Rancho land grant |
| Geographic setting | old terraces, fan remnants, erosional remnants and alluvial fans (mixed rock sources) |
| Latitude/Longitude | 35°, 0’, 04” North, 118°, 4’, 03” West |
| soil temperature | mean annual 15.6-19.4 ºC |
| mean annual precipitation | 200 - 360 mm |
| **A horizon parameter** |  |
| soil reaction | moderate alkaline, pH 8.0 |
| water content | moist from late October/November until late April or early May |
| organic matter | 1-2% |
| clay percentage | 15-35% |
| Sodium Adsorption Ratio | nd |
| Electrical conductivity | nd |
| **Chanac** |  |
| Soil survey area | Kern County (NE, NW, and SE part), San Luis Obispo County, Paso Robles Area, Carrizo Plain, Los Padres National Forest area |
| Sites investigated in this study | Ant Hill Oil Field, across CALM (map unit 130, 131) |
| Taxonomic class | fine-loamy, mixed superactive, thermic Calci Haploxerepts |
| Typical Pedon | sandy clay loam, loam, or clay loam, rangeland |
| vegetation | wild oats, red brome, other annual grasses, and filaree |
| Type Location | Kern County, Tejon Hills, Western foothills of Tehachapi mountains |
| Geographic setting | old stream terraces, formed from mixed alluvium of middle or lower Pliocene non-marine origin |
| Latitude/Longitude | 35°, 0’, 14” North, 118°, 4’, 17” West |
| soil temperature | mean annual 14-20 ºC |
| mean annual precipitation | 229-305 mm |
| **A horizon** |  |
| soil reaction | slightly alkaline, pH 7.5 |
| water content | nd |
| organic matter | nd |
| clay percentage | 18-35% |
| Sodium Adsorption Ratio | nd |
| Electrical conductivity | nd |
| *nd = not determined  **A horizon= the uppermost zone of soil, containing humus; topsoil | |

| **Table S2.** Extend of STH-vegetation class in our ROI between 2008 and | | | | |
| --- | --- | --- | --- | --- |
| 2011 based on satellite imagery. | | | |  |
|  | **Class area (km^2^)** | | **Percent area covered** | |
| **Year** | **P = 0.9** | **P = 0.95** | **P = 0.9** | **P = 0.95** |
| **2008** | 3740.7771 | 3429.1512 | 35.228% | 32.293% |
| **2009** | 4431.2535 | 4194.2268 | 41.731% | 39.498% |
| **2010** | 4609.3851 | 3951.1521 | 43.421% | 37.220% |
| **2011** | 2977.1460 | 2761.8525 | 28.045% | 26.017% |
